# Association of common *ATM *variants with familial breast cancer in a South American population

**DOI:** 10.1186/1471-2407-8-117

**Published:** 2008-04-23

**Authors:** Patricio González-Hormazábal, Teresa Bravo, Rafael Blanco, Carlos Y Valenzuela, Fernando Gómez, Enrique Waugh, Octavio Peralta, Waldo Ortuzar, Jose M Reyes, Lilian Jara

**Affiliations:** 1 Human Genetics Program, Institute of Biomedical Sciences (ICBM), School of Medicine, University of Chile, Chile; 2 National Cancer Society (Corporacion Nacional del Cancer, -CONAC-), Santiago, Chile; 3 Clínica Santa María, Santiago, Chile; 4 Department of Gynaecology and Obstetrics, School of Medicine, University of Chile, Chile; 5 Hospital Clinico de la Universidad de Chile, Chile; 6 Clínica Las Condes, Santiago, Chile

## Abstract

**Background:**

The *ATM *gene has been frequently involved in hereditary breast cancer as a low-penetrance susceptibility gene but evidence regarding the role of *ATM *as a breast cancer susceptibility gene has been contradictory.

**Methods:**

In this study, a full mutation analysis of the *ATM *gene was carried out in patients from 137 Chilean breast cancer families, of which 126 were *BRCA1/2 *negatives and 11 *BRCA1/2 *positives. We further perform a case-control study between the subgroup of 126 cases *BRCA1/2 *negatives and 200 controls for the 5557G>A missense variant and the IVS38-8T>C and the IVS24-9delT polymorphisms.

**Results:**

In the full mutation analysis we detected two missense variants and eight intronic polymorphisms. Carriers of the variant IVS24-9delT, or IVS38-8T>C, or 5557G>A showed an increase in breast cancer risk. The higher significance was observed in the carriers of IVS38-8T>C (OR = 3.09 [95%CI 1.11–8.59], p = 0.024). The IVS24-9 T/(-T), IVS38-8 T/C, 5557 G/A composite genotype confered a 3.19 fold increase in breast cancer risk (OR = 3.19 [95%CI 1.16–8.89], p = 0.021). The haplotype estimation suggested a strong linkage disequilibrium between the three markers (D' = 1). We detected only three haplotypes in the cases and control samples, some of these may be founder haplotypes in the Chilean population.

**Conclusion:**

The IVS24-9 T/(-T), IVS38-8 T/C, 5557 G/A composite genotype alone or in combination with certain genetic background and/or environmental factors, could modify the cancer risk by increasing genetic inestability or by altering the effect of the normal DNA damage response.

## Background

The incidence of breast cancer in Chile is still increasing but the mortality rate in the last decade has remained constant. Early detection contributes to mortality reduction and genetic testing may identify high-risk individuals. Mutations in *BRCA1 *and *BRCA2 *genes (*BRCA1/2*) have been identified as high penetrance alleles. However, these alleles explain only a small fraction of familial breast cancers [1,2]. We have previously screened for germ line mutations in *BRCA1/2*, 64 Chilean families with several cases of breast and/or ovarian cancer [3]. Only 15.6% of these presented mutations in one of these two genes. The most widely accepted model proposes that familial breast cancer susceptibility is a consequence of a small number of mutations in *BRCA1/2 *and a much larger variability in ethnic-specific genes of moderate and/or low penetrance [4]. The Ataxia-Telangiectasia Mutated gene (*ATM*) has been frequently involved in hereditary breast cancer as a low-penetrance susceptibility gene. The ATM kinase has an essential role in maintaining genomic integrity. It is a key activator of the cellular responses to DNA double-strand breaks [5]. Individuals heterozygous for *ATM *mutations have been reported to have an increased risk for female breast cancer. A number of studies have searched for germ line *ATM *mutations in breast cancer cases and/or compared the frequency of common *ATM *variants among breast cancer cases to population controls, but evidence regarding the role of *ATM *as a breast cancer susceptibility gene has been contradictory [6,7]. Recently, large epidemiological and molecular studies have finally provided conclusive evidence that *ATM *mutations that cause ataxia-telangiectasia are breast cancer susceptibility alleles [6,8]. There was no evidence that other classes of *ATM *variants confer a risk of breast cancer [8]. A common *ATM *variant IVS38-8T>C in cis with the 5557G>A *ATM *variant, has been suggested to be associated with bilateral breast cancer [9]. The 5557G>A variant has previously been reported in the homozygous state to associate with enhanced clinical radiosensitivity in breast cancer patients [10-12].

In this study, we screened the entire coding region and exon-intron boundaries of the *ATM *gene from 137 Chilean familial breast cancer patients. We further evaluated the 5557G>A missense variant and the IVS38-8T>C and the IVS24-9delT polymorphisms, for breast cancer risk in a subgroup of 126 familial breast cancer cases without *BRCA1/2 *mutations and in 200 healthy controls.

## Methods

### Subjects

A total of 137 breast cancer patients (one case per family) belonging to 137 high risk Chilean families were selected from the files of the Metropolitan Santiago National Health Service, National Cancer Society (Corporación Nacional del Cancer -CONAC-) and the Arturo Lopez Perez Foundation. Table 1 shows the specific characteristics of the selected families according to the inclusion criteria. All of the families participating in the study self reported Chilean ancestry several generations ago, after extensive interviews with several members of each family pertaining to different generations. In the selected families, 13.9% (19/137) presented cases of bilateral breast cancer; and 7.3% (10/137) presented cases of both breast and ovarian cancer. In the breast cancer group, the mean age of diagnosis was 44,6 years and 67,9% had age onset <50 years. Breast cancer was verified by the original pathology report for all probands. The 137 index cases (one case per family) were tested for *BRCA1 *and *BRCA2 *mutations as described elsewhere [3], of which 126 were *BRCA1/2 *negatives and 11 *BRCA1/2 *positives.

**Table 1:** Selection criteria and clinical characteristics of the families included in this study.

Selection Criteria	FAMILIES
2 family members with breast cancer	29
2 family members with breast cancer, onset before age 40 in one	18
≥ 2 family members with breast cancer, bilateral in one	15
≥ 3 family members with breast cancer	32
≥ 3 family members with breast cancer, at least one with onset before age 40	26
3 family members with breast cancer, one male cancer	2
≥ 3 family members with a combination of breast and ovarian cancer	3
≥ 2 family members with breast cancer, one with both breast and ovarian cancer	2
Single affected individual with breast cancer < age 31	10
Breast cancer in male only	2

**Total**	**137**

Two hundred Chilean females used as controls were recruited from the files of the CONAC, matched by age and ethnic background with cases. Cases and controls belonged to the same geographical area and the same socioeconomic strata. Controls were interviewed and informed as to the aims of the study. Control DNA samples were taken from unrelated individuals with no personal or familial history of breast or other cancer who gave their consent for anonymous testing. Samples were obtained under considerations of all ethical and legal requirements.

This study was approved by the Institutional Review Board of the School of Medicine of the University of Chile. Informed consent was obtained from all the participants.

### Mutation analysis

Genomic DNA of the 137 breast cancer cases was extracted from peripheral blood lymphocytes obtained according to the method described by Chomczynski and Sacchi [13]. The whole coding sequence and exon-intron boundaries of the *ATM *gene were amplified by polymerase chain reaction (PCR). Primers were designed using PRIMER3 software (See Additional file 1: Primers for *ATM *mutation analysis and see Availability and requirements for URL). The fragments obtained were analyzed for sequence variants using conformational sensitive gel electrophoresis (CSGE) [14]. Amplified samples were denaturated at 95°C for 5 minutes and 65°C for 30 minutes to generate heteroduplex. The products were diluted 1:2 in sucrose buffer and loaded in a partially denaturing MDE^® ^gel (Cambrex, UK) at constant power of 7 W during different time periods depending on the size of the fragment. Gels were silver-stained and dried on a vacuum gel dryer. All sequence variants detected by CSGE were identified by reamplification of the original DNA sample and direct sequencing was performed in an ABI Prism 310 automated fluorescence-based cycle sequencer and a Rhodamine dye terminator system (Perkin Elmer – Applied Biosystems, Foster City, CA). The CSGE assay is technically simple and has a high sensitivity for the detection of mutations. The main factors influencing sensitivity are the gel matrix and the identity of base mismatch [15,16]. MDE matrix is a polyacrylamide-like matrix that has a high sensitivity to DNA conformational differences. Separation on a MDE gel provides superior results when compared with standard polyacrilamide gels [17,18].

A polymerase chain reaction – restriction fragment length polymorphism (PCR-RFLP) assay was used to genotype IVS24-9delT, IVS38-8T>C and 5557G>A variants in the subgroup of 126 cases *BRCA1/2 *negatives and in the 200 controls. PCR products were digested overnight with restriction enzymes (New England Biolabs, MA, USA) according to the manufacturer protocols and analyzed in 2% agarose gel electrophoresis. Primers, restriction enzymes and the length of digested fragments are shown in Table 2. Each sample of cases and controls for IVS24-9delT, IVS38-8T>C and 5557G>A variants were amplified and digested in two different occasions. We used PCR-RFLP for genotyping IVS24-9delT, IVS38-8T>C and 5557G>A variants because CSGE only permits to detect heterozygous. All the heterozygotes for each variant were confirmed by sequencing and 100% concordance was found between the RFLP and CSGE genotyping.

**Table 2:** Primers, restriction enzymes and fragment lengths for IVS24-9delT, 5557G>A and IVS38-8T>C variants

Variant	Primer secuence (1)	Anealing temperature	Restriction enzyme	Fragment lengths
IVS24-9delT	F 5' ACTAAGCTGCTGGTCTGAAC 3' R 5' GTCCTGGAACAATCTTAAAGC 3'	48°C	*FnuH *I	T allele : 176 bp + 24 bp (-T) allele: 199 bp
IVS38-8T>C	F 5' ATGGTAATGGCCTAGACTGG 3' R 5' ATCCAAGTTTGCAGGGGTTG 3'	58°C	*Rsa *I	T allele: 295 bp + 148 bp C allele: 260 bp + 148 bp + 35 bp
5557G>A	F 5' TAATATGTCAACGGGGCATG 3' R 5' ATTTCTCCATGATTCATTTGGAT 3'	52°C	*Rsa *I	G allele: 156 bp + 23 bp A allele: 179 bp

(1) Underlined base indicates a mismatch to create the restriction site

### Statistical analysis

The Hardy-Weinberg equilibrium assumption was assessed using standard maximum likelihood methods. Fisher's exact-test was used to asses the association of alleles and/or genotypes between cases and controls, *p *< 0,05 was used as the criterion of significance. The odds ratio (OR) and their 95% confidence interval (CI) were calculated to estimate the strength of the association between cases and controls. Haplotype estimation was carried out using UNPHASED software which uses a maximum likelihood approach [19].

## Results

Table 3 shows the sequence variants found in the *ATM *gene in the 137 patients. We detected two missense mutations and eight intronic polymorphisms. The most frequent variants were: IVS4+36insAA (46%); IVS48-69insATT (50.5%); IVS24-9delT (20.6%) and 5557G>A (20.6%). All the polymorphisms and the aminoacid substitutions detected in the present study have been previously described in different populations such as Caucasians, Africans and Amerindians [20-22]. Of the detected variants, eight were intronic polymorphisms, the majority of which were located far of the donor or acceptor splice sites. Then, the probability that they represent risk variants is very low. Thus, we considered not necessary to study the frequency of the following variants in controls: IVS4+36insAA, IVS17-56G>A, IV25-12insA, IVS38-15G>C, IVS47-65G>C, and IVS48-69insATT.

**Table 3:** *ATM *variants found in familial breast cancer cases.

Intron/exon	Variant	HUGO (1) nomenclature	rsID (2)	Effect	Heterozygous frecuency	Heterozygous frecuency dbSNP database	Described by
4	IVS4+36insAA	c.72+36insAA	rs2066734	Unknown	46,0%	41.9% (3)	[20]
6	378T>A	c.378T>A	rs2234997	p.D126E	0,8%	8.7% (4)	[22]
17	IVS17-56G>A	c.2377-56G>A	rs672655	Unknown	46,8%	43.6% (3)	[20]
24	IVS24-9delT	c.3249-9delT	rs3218698	Unknown	20,6%	13,3% (5)	[20]
25	IVS25-12insA	c.3366-12insA	rs4987984	Unknown	49,2%	48.2% (3)	[22]
38	IVS38-15G>C	c.5320-15G>C	rs3092828	Unknown	0,8%	1.0% (6)	[22]
38	IVS38-8T>C	c.5320-8T>C	rs3092829	Unknown	8,7%	4,5% (4)	[20]
38	5557G>A	c.5557G>A	rs1801516	p.D1853N	20,6%	10,3% (5)	[20]
47	IVS47-65G>C	c.6516-65G>C	rs4988104	Unknown	1,7%	2.4% (7)	-
48	IVS48-69insATT	c.6751-69insATT	rs3212322	Unknown	49,2%	43.2% (3)	[21]

(1) HUGO: Human Genome Organization.

(2) rs numbers are from human single nucleotide polimorphisms database (dbSNP)

(3) Estimated frequency in NCBI:NIHPDR sample (90 individuals from North American population). .

(4) Estimated frequency in SNP500CANCER:HISP1 sample (23 individuals from Hispanic population). .

(5) Estimated frequency in NIHPDR sample (450 unselected for ethnicity) .

(6) Estimated frequency in OEFNER:autosome sample (97 individual unselected for ethnicity). .

(7) Estimated frequency in EGP_SNPS:PDR90 sample (90 individual unselected for ethnicity). .

In the current investigation, we performed a case-control study between the subgroup of 126 cases *BRCA1/2 *negative and 200 controls, for the 5557G>A missense variant and for the IVS38-8T>C and IVS24-9delT polymorphisms. Heikkinen et al. [9] proposed a cancer risk-modifying effect for the *ATM *5557G>A – IVS38-8 T>C composite allele. The IVS24-9delT was studied because it is located in the acceptor splice site of intron 24 and for its higher frequency in cases. We analyzed the allelic, genotype and composite genotype frequencies for the variants mentioned above and compared them with controls. These variants were in Hardy-Weinberg equilibrium in the studied groups. The frequency of 5557A allele (allele analysis) was 0.10 in *BRCA1/2 *negative cases versus 0,06 in controls (p = 0,056, OR = 1.67 [95% CI 0.94–2.92]). Nevertheless, the frequency of 5557A allele carriers (genotype analysis) was 0.21 in cases versus 0.13 in controls (p = 0.048, OR = 1.74 [95%CI 0.96–3.16]) (Table 4). Similar results were observed for the allelic and genotype frequencies of the IVS24-9delT polymorphism (Table 4). With respect to IVS38-8T>C polymorphism, the IVS38-8C allele frequency was higher in *BRCA1/2 *negative cases (0.05) than in healthy controls (0.01) and the difference was significant (p = 0.025, OR = 3.00 [95%CI 1.09–8.21]) (Table 4). Moreover, we observed a higher frequency of IVS38-8C allele carriers in cases versus controls, being the difference statistically significant (p = 0.024, OR = 3.09 [95%CI 1.11–8.59]). Therefore, each of the genotypes may confer an increase in breast cancer risk. Nevertheless the higher significance was observed in the carriers of the IVS38-8T>C. With respect to the 11 *BRCA1/2 *positive index cases, none of them carried the analyzed variants.

**Table 4:** Genotype and allele frequencies of IVS24-9delT, IVS38-8T>C and 5557G>A *ATM *variants in *BRCA1/2 *negative breast cancer cases and controls.

***ATM *variant**	**BC cases (%) (n = 126)**	**Controls (%) (n = 200)**	**p value (a)**	**OR [95%CI]**
**IVS24-9delT**				

T/T	100 (79.4%)	174 (87.0%)	1.000	1.00
T/(-T)	26 (20.6%)	26 (13.0%)	0.048	1.74 [0.96–3.16]
(-T)/(-T)	0 (0%)	0 (0%)	-	-
T allele	226 (0.90)	374 (0.94)	1.00	1.00
(-T) allele	26 (0.10)	26 (0.06)	0.056	1.67 [0.94–2.92]

**IVS38-8T>C**				

T/T	115 (91.3%)	194 (97.0%)	1.000	1.00
T/C	11 (8.7%)	6 (3.0%)	0.024	3.09 [1.11–8.59]
C/C	0 (0%)	0 (0%)	-	-
T allele	241 (0.95)	394 (0.99)	1.000	1.00
C allele	11 (0.05)	6 (0.01)	0.025	3.00 [1.09–8.21]

**5557G>A**				

G/G	100 (79.4%)	174 (87.0%)	1.000	1.00
G/A	26 (20.6%)	26 (13.0%)	0.048	1.74 [0.96–3.16]
A/A	0 (0%)	0 (0%)	-	-
G allele	226 (0.90)	374 (0.94)	1.000	1.00
A allele	26 (0.10)	26 (0.06)	0.056	1.67 [0.94–2.92]

BC: breast cancer.

(a) Fisher exact test.

Table 5 shows the composite genotype analysis for IVS24-9delT, IVS38-8T>C and 5557G>A. In accordance with the findings of Heikkinen et al. [9] and Langholz et al. [23], we also observed that IVS38-8T>C carriers were also carriers for 5557G>A in *BRCA1/2 *negative cases and controls. The IVS24-9 T/(-T), IVS38-8 T/C, 5557 G/A composite genotype showed a higher frequency in cases (8,7%) compared to controls (3.0%) (p = 0.021, OR = 3.19 [95%CI 1.16–8.89]) (Table 5). Thus, this composite genotype confers a 3.19 fold increase in breast cancer risk. In addition, the frequency of T/(-T), T/T, G/A composite genotype did not differ between *BRCA1/2 *negative cases (11.9%) versus controls (10.0%) (p = 0.289, OR = 1.31 [95%CI 0.63–2.66]). Table 5 also shows that the analyzed variants are highly correlated in relation to the risk for breast cancer. All the IVS38-8T>C heterozygotes were also IVS24-9delT and 5557G>A heterozygotes, both in cases (11/126) and controls (6/200). The difference is given by IVS24-9delT heterozygotes that are also associated to IVS38-8 T/T (15/126 in cases and 20/200 in controls), but not to 5557 G/G (0/126 cases, 0/200 controls), and 5557G>A heterozygotes that are not associated to IVS24-9 T/T (0/126 cases, 0/200 controls), and are associated to IVS38-8 T/T (15/126 cases, 20/200 controls). This fact raises the possibility for these variants to be in strong linkage disequilibrium.

**Table 5:** Composite genotype frequencies for IVS24-9delT, IVS38-8T>C and 5557G>A *ATM *variants in *BRCA1/2 *negative breast cancer cases and controls.

**Composite genotype**	**BC cases (n = 126)**	**Controls (n = 200)**	**p value (a)**	**OR [95%CI]**
**IVS24-9delT, IVS38-8T>C**				

T/T, T/T	100 (79.4%)	174 (87.0%)	1.000	1.00
T/(-T), T/T	15 (11.9%)	20 (10.0%)	0.289	1.31 [0.63–2.66]
T/(-T), T/C	11 (8.7%)	6 (3.0%)	0.021	3.19 [1.16–8.89]

**IVS38-8T>C, 5557G>A**				

T/T, G/G	100 (79.4%)	174 (87.0%)	1.000	1.00
T/T, G/A	15 (11.9%)	20 (10.0%)	0.289	1.31 [0.63–2.66]
T/C, G/A	11 (8.7%)	6 (3.0%)	0.021	3.19 [1.16–8.89]

**IVS24-9delT, 5557G>A**				

T/T, G/G	100 (79.4%)	174 (87.0%)	1.000	1.00
T/(-T), G/A	26 (20.6%)	26 (13.0%)	0.048	1.74 [0.96–3.16]

**IVS24-9delT, IVS38-8T>C, 5557G>A**				

T/T, T/T, G/G	100 (79.4%)	174 (87.0%)	1.000	1.00
T/(-T), T/T, G/A	15 (11.9%)	20 (10.0%)	0.289	1.31 [0.63–2.66]
T(/-T), T/C, G/A	11 (8.7%)	6 (3.0%)	0.021	3.19 [1.16–8.89]

BC: breast cancer.

(a) Fisher exact test.

Although phase for alleles at these 3 variants could not be determined directly from the screening data, haplotypes where constructed from genotype data using UNPHASED software, which uses a maximum-likelihood aproach. The total number of haplotypes with the three markers are eight, but in the cases and controls we observed only three which correspond to wildtype (IVS24-9 T – IVS38-8 T – 5557 G); IVS24-9 (-T) – IVS38-8 T – 5557 A; and IVS24-9 (-T) – IVS38-8 C – 5557 A haplotypes. The haplotype estimation suggested a strong linkage disequilibrium between the three markers (coefficient of linkage disequilibrium, D' = 1), a result which had previously been suggested by composite genotype analysis.

## Discussion

Heterozygous individuals for germline *ATM *mutations have been reported to have an increased risk for malignancy, in particular, female breast cancer. In this study we detected a low *ATM *diversity in breast cancer women, and this variation was minor in the coding region with respect to noncoding regions. This result is in accordance to Thorterson et al. [22], which established that the nucleotide diversity of *ATM *in the coding and non coding region have a ratio of 1:7.5, probably due to selective pressure for maintaining the protein sequence. The *ATM *variations in this study were also minor with respect to those informed by Tapia et al. [24]in Chilean women with hereditary breast cancer. Probably, this may be due to the ethnic origin of the families included. In Tapia et al. study [24], 28% of the families were of European ancestry, probably biasing the results of this study. In our study, all of the families were of Chilean ancestry several generations ago.

In the present study all the polymorphisms and the aminoacid substitutions detected have been previously described in other populations of different ethnic origins, including the *ATM *missense variant 5557G>A and the IVS24-9delT and IVS38-8T>C polymorphisms.

Some previous studies have proposed a phenotypic effect for the common *ATM *missense variation 5557G>A in exon 39 [10-12]. Heikkinen et al. [9], in their mutation analysis of the *ATM *gene in patients from 121 Northern Finnish breast or breast-ovarian cancer families reported that the haplotype composed of the alleles 5557A and IVS38-8C was significantly associated with bilateral breast cancer. In a later report, Langholz et al. [23] in the WECARE Study population did not find that the haplotype of 5557G>A and IVS38-8T>C confered an increased risk to women with bilateral breast cancer when compared with women with unilateral breast cancer. Nevertheless, the WECARE Study is population-based and unselected for family history. Tommiska et al. [25] evaluated the 5557G>A and IVS38-8T>C variants in an extensive analysis that included familial breast cancer cases and healthy controls of Southern Finland. In their study neither 5557G>A nor IVS38-8T>C or any haplotype containing these variants, was associated with breast cancer risk or bilateral cancer, but in familial breast cancer cases there was a higher frequency of IVS38-8T>C carriers than in controls (8.1% and 5.6%, respectively). Tapia et al. [24] in a study in Chilean women with hereditary breast cancer found a positive association of 5557G>A (OR = 2.52, p = 0.008), when analyzed alone and in combination with the IVS24-9delT intronic variant (OR = 3.97, p = 0.0003). In our case-control study of the IVS38-8T>C, 5557G>A and IVS24-9delT variants, the frequency of IVS38-8C allele was higher in *BRCA1/2 *negative cases (0.05) than in controls (0.01) and the difference was significant (p = 0.025, OR = 3.00 [95%CI 1.09–8.21]) (Table 4). The database (dbSNP rs3092829) reported for IVS38-8C allele frequencies of 0.023 in the Hispanic population, 0.016 in Caucasians and 0.01 in Europeans which are in accordance with those of our control group. The frequency of the 5557A allele in controls is also similar to those published in the database (dbSNP rs1801516). Our results with respect to IVS38-8T>C and 5557G>A allele control frequencies differ from those reported by Tapia et al. [24], which selected the control sample from a blood bank of Santiago, Chile. In our study controls were interviewed, age matched with cases, and correspond to individuals with no personal or familial history of breast and other cancer. Cases and controls were from the same ethnic group, and from the same geographic area. The major problem in case-control studies is ensuring a good match between their genetic background, so that any genetic difference between them is related to the disease under study and not to biased sampling [26]. This may be the reason why in the Tapia et al. study [24] controls showed no significant differences with cases in the allele frequencies of these markers.

In our study the carriers of the variant IVS24-9delT, or IVS38-8T>A, or 5557G>A (genotype frequencies) showed an increase in breast cancer risk, although the statistical significance was higher for the variant IVS38-8T>C (OR = 3.09 [95%CI 1.11–8.59] p = 0.024) (Table 4).

We also analyzed the effect of the composite genotypes in breast cancer risk. In accordance with the findings of Heikkinen et al. [9] and Tommiska et al. [25] in the Finnish population, and Langholz et al. [23] in the WECARE sample, we observed that all the carriers of IVS38-8C allele were also carriers of 5557A allele. Then, it is possible that IVS38-8T>C occurs in combination with 5557G>A. We observed, both in cases and controls, only three of the 27 possible composite genotypes, and of these the triheterozygote composite genotype showed a higher frequency in cases (8.7%) than controls (3.0%), being the difference statistically significant (OR = 3.19 [95%CI 1.16–8.89], p = 0.021) conferring an increased risk among women with familial breast cancer. The composite genotype T/(-T), T/T, G/A presented a similar frequency in cases (11.9%) and in controls (10.0%) (Table 5). In agreement with Heikkinen's report [9], our results could indicate that the heterozygous condition for IVS38-8T>C and 5557G>A may be responsible of the association between T/(-T), T/C, G/A composite genotype and an increased risk of familial breast cancer. Nevertheless, we cannot rule out the possibility that T/(-T), T/C, G/A constitutes a risk composite genotype. Another possibility is that this composite genotype in combination with certain genetic background and/or environmental factors, could modify the cancer risk by increasing genetic inestability or by altering the effect of the normal DNA damage response. The present observations need to be confirmed by additional case-control and functional studies.

We observed a strong linkage disequilibrium between the three analyzed markers. Therefore, of the three haplotypes detected in the cases and control samples some of theese may be founder haplotypes, given that the contemporary Chilean population stems from the admixture of Amerindian peoples with the Spanish settlers (European Caucasian). The relationship between ethnicity, Amerindian admixture, genetic markers, and socioeconomic strata has been extensively studied in Chile [27-29]. Moreover, it has been reported a low prevalence of breast cancer in those regions of the country where the Amerindian population is concentrated and represents demographically a substancial proportion of the people of such regions.

With the exception of the studies performed in Northern and Southern Finnish populations, there are no reports for other European populations were the association between familial breast cancer with the IVS24-9delT, IVS38-8T>C and 5557G>A variants has been analyzed. Therefore, probably the association reported in our study may also exist in other populations with a different ethnic background, such as other European, Asian or Amerindian populations.

With respect to the Amerindian populations a consensus among anthropologists was reached in the sense that Amerindians derived from Asians which, moving from Asia, crossed the Bering Land Bridge [30]. Then, we also raise the hypothesis that the haplotype IVS24-9(-T) – IVS38-8C – 5557A may be a risk haplotype in some contemporary South American populations that stem from the admixture of Amerindian peoples with the Spanish settlers. Up to date, there are no reports of these variants in Asian and Spanish populations.

## Conclusion

The findings of our study were: a) we determined that the *BRCA1/2 *negatives cancer patients that were carriers of the IVS24-9delT, IVS38-8T>C or 5557G>A showed an increase in brest cancer risk; b) we observed that all the carriers of IVS38-8C allele were also carriers of IVS24-9delT and 5557A alleles, a finding which suggests that IVS38-8T>C occurs in combination with the latter variants; and c) we established that the IVS24-9 T/(-T), IVS38-8 T/C, 5557G/A composite genotype confers a 3,19 fold increase in breast cancer. The most important finding and contributions of this study is that a triheterozygous composite genotype confers an increase in the risk for breast cancer. Probably this composite genotype exists in the structure of the Chilean population which may also exists in other South American poulations.

## List of abbreviations

*BRCA1/2*: *BRCA1 *and *BRCA2 *genes; OR: Odds Ratio; CI: Confidence Interval.

## Competing interests

The authors declare that they have no competing interests.

## Availability and requirements

PRIMER3 software: 

## Authors' contributions

PGH, RB and LJ performed analysis and prepared the manuscript. CYV performed statistical analysis. FG, EW, OP, WO and JMR selected familial breast cancer cases from different oncology services. TB was responsible of controls selection.

## Pre-publication history

The pre-publication history for this paper can be accessed here:



## Supplementary Material

Additional file 1 supplementary_table_S1. Primers for *ATM *mutation analysis Click here for file
