# Supplementary material for: Association of common ATM variants with familial breast cancer in a South American population
Source: BMC Cancer. 2008 Apr 23;8:117. doi: 10.1186/1471-2407-8-117 (PMC2386480; doi:10.1186/1471-2407-8-117)
Supplement: Additional file 1 — supplementary_table_S1. Primers for ATM mutation analysis [file 1471-2407-8-117-S1.doc]

**Table S1. Primers for *ATM* mutation analysis**

| **EXON** | **Forward primer (5’ – 3’)** | **Reverse primer (5’ – 3’)** | **Lenght (bp)** | **3' intron boundary (bp)** | **5' intron boundary (bp)** | **Anealling temperature (Cº)** |
| --- | --- | --- | --- | --- | --- | --- |
| 3 & 4 | CCAGAATGTGCCTCTAATTG | GATAAATGTTAAGACTTACACAC | 536 | 126 | 73 | 54 |
| 5 | TTTGTGATGGCATGAACAGC | ACTTATGCAACAGTTAAGTCC | 333 | 64 | 82 | 56 |
| 6 | AGTTGCCATTCCAAGTGTCT | ACTGTTAAACTGTCAGGTCAC | 349 | 49 | 94 | 58 |
| 7 | CTTTTTCTGTATGGGATTATGG | GTGTTGGACTTGGTTGTCTC | 426 | 102 | 158 | 58 |
| 8 | GTCAGTGTGAAGTAATGCTG | CAACCAGAGAAATCCAGAGG | 531 | 157 | 95 | 58 |
| 9 | TGGGAGCTAGCAGTGTAAAC | TAAAAGCCCAAAATGCCCAG | 304 | 44 | 56 | 54 |
| 10 | GTAGAGGTTGTGGTGATACG | GAGATGAAAGGATTCCACTG | 373 | 95 | 68 | 54 |
| 11 | AGAGTACTATGGAAATGATGG | TCTGTAAGTCAGACATAATGC | 533 | 106 | 13 | 54 |
| 12 | TAACTAAATGTATGTGCCAGG | GGCATCAAATAAGTGGAGAG | 365 | 73 | 56 | 54 |
| 13 | TTCTTTACATGGCTTTTGGTC | AAGATGCAGCTACTACCCAG | 237 | 32 | 68 | 54 |
| 14 | GCTAATACATATAAGGCAAAGC | CAATTTAGAGCCCTTTACTGC | 438 | 44 | 125 | 58 |
| 15 | TAGAGCTATCCAGGATATGC | GACGAGTTGATGGGTGCAG | 549 | 125 | 268 | 58 |
| 16 | ACTGTATGACTACGTGGAAC | ATGCCTGTAGTCCCAATTAC | 452 | 141 | 140 | 58 |
| 17 | AGCACAATGAAAGATGTACAG | GTGAGATGCATCCTTATTTAC | 372 | 169 | 71 | 58 |
| 18 | GAAGCCATCTTGAACATCTTTG | GCCTCTTATACTGCCAAATC | 277 | 17 | 46 | 52 |
| 19 | GTGAAGAGGAGGAAATTTGAG | AGGTTCATTTCTCAAGAAAGC | 397 | 48 | 107 | 58 |
| 20 & 21 | TTGTGATCTGCCTGCTTCAG | GCATTCGTATCCACAGATAG | 538 | 116 | 38 | 58 |
| 22 & 23 | TTCAGTGAGTTTTCTGAGTGC | GACATTCTACTGCCATCTGC | 413 | 47 | 37 | 58 |
| 24 | TGCTTTGGAAAGTAGGGTTTG | TATGGGATATTCATAGCAAGC | 249 | 53 | 36 | 58 |
| 25 | AAATCTGGAGTTCAGTTGGG | GTGCCACTCAGAAAATCTAG | 346 | 64 | 68 | 58 |
| 26 | GTTGTTTCTAGGTCCTACTC | AGGGGACTTGCTAAGTATTG | 337 | 76 | 51 | 58 |
| 27 | GTGGTATGTTCTAAGCTTTC | GGTTGGCTATGCTAGATAATG | 600 | 191 | 121 | 58 |
| 28 | TTTGAGCTGTCTTGACGTTC | AGGTGTCAACCAATAAACTTC | 254 | 45 | 52 | 58 |
| 29 | TGGAAGTTCACTGGTCTATG | AAGTAGATATATTCCCTGAGC | 442 | 50 | 224 | 52 |
| 30 | AGTGTATTTATTGTAGCCGAG | CTGGGCAATATGGTGAAAC | 532 | 43 | 249 | 58 |
| 31 | GATGCTGAACAAAAGGACTTC | GTACCAAGTGCTCTGTAATTG | 450 | 72 | 161 | 50 |
| 32 | TAAAAGCTGGGTATCTTAGAC | TATAGGCATGAGCCAATGTG | 461 | 122 | 133 | 54 |
| 33 | CATTGTAGGGTTTGCAGTGG | TAGATGAGAAATGAGGGTCTG | 443 | 151 | 118 | 58 |
| 34 | AAAGTGTTGTCTTCATGCTAG | TACAGGCAACAGAAAACATAC | 251 | 31 | 82 | 54 |
| 35 | CTCTTACCTATGACTCTACTG | GTATCATTCTCCATGAATGTC | 273 | 21 | 38 | 52 |
| 36 | ATAACTGGTGTACTTGATAGG | AACACATTCCCTGGATTTATG | 345 | 21 | 140 | 54 |
| 37 | AGGAAAGGTACAATGATTTCC | AACAGTTTGAGTGGGGGTGA | 348 | 39 | 91 | 58 |
| 38 | TAATATGTCAACGGGGCATG | TGCTTTTAGTGGGATTCCATC | 356 | 75 | 62 | 50 |
| 39 | TGGGAGACAGACACATAAAC | CATGTTAAAATTCAGCCGATAG | 248 | 64 | 54 | 50 |
| 40 | GATTTCTTGGTAGAGAGCAG | CCTTATTGAGACAATGCCAAC | 513 | 279 | 37 | 52 |
| 41 | GTATATGTATTCAGGAGCTTC | ATGGCATCTGTACAGTGTC | 238 | 45 | 65 | 50 |
| 42 & 43 | GAGTTGGGAGTTACATATTGG | GAGTATCCCTGAATGTTTAG | 526 | 81 | 136 | 48 |
| 44 | ACACCCAGCTGATATTTTGG | GTTTAGAATGAGGAGAGAGG | 345 | 124 | 32 | 50 |
| 45 | CATGTATATCTTAGGGTTCTG | TCTCTTCATCAATGCAAATCC | 270 | 37 | 86 | 48 |
| 46 | CATTTATTTCCCTGAAAACCTC | CTATTGGTAACAGAAAAGCTG | 237 | 39 | 35 | 54 |
| 47 | GTAGGATTATTTACAAGTTCTAG | TCTTTTTCCCTCAGGCTTTC | 468 | 127 | 63 | 54 |
| 48 | GAATGGTAGTTGCTGCTTTC | TTCATTCATTTCCCACATACC | 542 | 72 | 261 | 54 |
| 49 | GCAGTTGGGTACAGTCATGG | GATCTTGATGAAAAGATGAAGC | 242 | 39 | 47 | 54 |
| 50 | TGTTGGGCCACATTCAAAGC | GTTAAGCCGACCTTTAGAGC | 543 | 181 | 104 | 54 |
| 51 | CCCTGGGATAAAAACCCAAC | TTATGTGTAGAGCACTGGAC | 439 | 91 | 100 | 54 |
| 52 | CCCATTAGAAAGACCTTCAG | GGAATTAGAGAGAGAAAATGAG | 465 | 167 | 142 | 54 |
| 53 | CCACTGCAGTATCTAGACAG | GCCTTGAACCGATTTTAGATG | 380 | 89 | 91 | 58 |
| 54 | CCTCAAAGCAGTTGGCAAAG | CTCTACAGAGAGTAACACAGC | 432 | 217 | 35 | 58 |
| 55 | TCTGCTGACTATTCCTGCTTG | ATAAGCACACGGAAACTCTC | 449 | 55 | 270 | 58 |
| 56 | CACATCGTCATTTGTTTCTCTG | GACAAAATCCCAAATAAAGCAG | 347 | 92 | 70 | 58 |
| 57 | CTATTCTCAGATGACTCTGTG | TCACCCAACCAAATGGCATC | 254 | 53 | 43 | 58 |
| 58 | TCTCAAACATCTAGGCAGCA | CACTCACACACTTTCATTCTG | 529 | 195 | 143 | 58 |
| 59 | CTGTCCAGACTGTTAGCTTC | TAATGAAGATGGGTTGGTTAC | 454 | 126 | 121 | 58 |
| 60 | CCAAGTCAGTGGTCTTAATTG | TGGTAGGCAAACAACATTCC | 250 | 69 | 53 | 58 |
| 61 | AGTTCACATTCTAACTGGAAAG | GCCCAGCCCATGTAATTTTG | 248 | 51 | 40 | 58 |
| 62 | AAAGATACGTTGACAACATTGG | GTGCTCTTCACATCAGTGAC | 213 | 40 | 67 | 58 |
| 63 & 64 | TGATACTGGTTCTACTGTTTC | TTCTAAAGGCTGAATGAAAGG | 545 | 57 | 19 | 54 |
